# Supplementary material for: Integrating long-range connectivity information into de Bruijn graphs
Source: Bioinformatics. 2018 Mar 15;34(15):2556–65. doi: 10.1093/bioinformatics/bty157 (PMC6061703; doi:10.1093/bioinformatics/bty157)
Supplement: Supplementary Data [file bty157_supp_proof_kvg.pdf]

Integrating long-range connectivity information into de Bruijn graphs: supplemental

Isaac Turner, Kiran V Garimella, Zamin Iqbal, Gil McVean

1 Choosing a cleaning threshold

Given a distribution of coverage *covg* (k-mer counts or link counts) and a user specified false negative rate (FNR) (default is $FNR = \frac{1}{1000}$), we fit the following model to pick a threshold T, such that fewer than FNR of elements with coverage T are due to error. Elements with coverage less than T are assumed to be due to error.

We assume k-mers that occur three or fewer times are due to error and fit a Gamma-Poisson mixture distribution to the erroneous coverage ($errcovg$). We then find the lowest level of coverage T such that $errcovg(T)/covg(T) < FNR$. Specifically, we model the probability of seeing an erroneous k-mer with coverage x, $p(x)$, as a Poisson distribution with mean drawn from a gamma distribution:

$$p(x|\alpha,\beta) = \int \frac{\beta^\alpha}{\Gamma\alpha}\mu^{\alpha-1}e^{-\beta\mu}.\frac{e^{-\mu}\mu^x}{x!}d\mu \tag{1}$$

$$= \frac{\beta^\alpha}{\Gamma(\alpha)}\frac{1}{x!}\int \mu^{\alpha+x-1}e^{-\mu(1+\beta)}d\mu \tag{2}$$

$$= \frac{\beta^\alpha}{\Gamma(\alpha)}\frac{1}{x!}\frac{\Gamma(\alpha+x)}{(1+\beta)^{(\alpha+x)}} \tag{3}$$

(3) derived from (1) using the identity for the Gamma Function $\Gamma(t) = \int x^{t-1}e^{-x}dx$ and setting $x = \mu(1+\beta)$, $t = \alpha + x$, which gives:

$$\Gamma(\alpha+x) = \int (\mu(1+\beta))^{\alpha+x-1}e^{-\mu(1+\beta)}d(\mu(1+\beta)) \tag{4}$$

$$\Gamma(\alpha+x) = \int \mu^{\alpha+x-1}(1+\beta)^{\alpha+x-1}e^{-\mu(1+\beta)}d(\mu(1+\beta)) \tag{5}$$

$$\frac{\Gamma(\alpha+x)}{(1+\beta)^{\alpha+x}} = \int \mu^{\alpha+x-1}e^{-\mu(1+\beta)}d\mu \tag{6}$$

Since observed coverage of errors is conditional on having seen a k-mer we assume $p(k|k > 0, \mu) \sim p(k-1|k > 0, \frac{\mu}{e^{-\mu}-1} - 1)$. Assume k-mer with coverage ≤ 3 are due to error. Use this to estimate $\hat{\alpha}$ using (3):

$$p(1)/p(0) = \frac{\left(\frac{\beta^\alpha}{\Gamma(\alpha)}\frac{1}{1!}\frac{\Gamma(\alpha+1)}{(1+\beta)^{(\alpha+1)}}\right)}{\left(\frac{\beta^\alpha}{\Gamma(\alpha)}\frac{1}{0!}\frac{\Gamma(\alpha)}{(1+\beta)^{\alpha}}\right)} \tag{7}$$

$$= \frac{\Gamma(\alpha+1)(1+\beta)}{\Gamma(\alpha)} \tag{8}$$

$$p(2)/p(1) = \frac{\Gamma(\alpha+2)(1+\beta)}{2\Gamma(\alpha+1)} \tag{9}$$

$$\frac{p(2)/p(1)}{p(1)/p(0)} = \frac{\left(\frac{\Gamma(\alpha+2)(1+\beta)}{2\Gamma(\alpha+1)}\right)}{\left(\frac{\Gamma(\alpha+1)(1+\beta)}{\Gamma(\alpha)}\right)} \tag{10}$$

$$= \frac{\Gamma(\alpha+2)\Gamma(\alpha)}{2\Gamma(\alpha+1)^2} \tag{11}$$

Using $covg(x)$ = Number of k-mers with coverage x, estimate $\hat{\alpha}$ by finding value that minimises the absolute difference between distribution and coverage data.

$$\hat{\alpha} = \min_{\alpha}\left|\frac{p(2)/p(1)}{p(1)/p(0)} - \frac{covg(3)/covg(2)}{covg(2)/covg(1)}\right| \tag{12}$$

$$\hat{\alpha} = \min_{\alpha}\left|\frac{\Gamma(\alpha+2)\Gamma(\alpha)}{2\Gamma(\alpha+1)^2} - \frac{covg(3)\times covg(1)}{covg(2)^2}\right| \tag{13}$$

Now find $\hat{\beta}$ using $\hat{\alpha}$:

$$p(1)/p(0) = covg(2)/covg(1)$$

$$\frac{\Gamma(\hat{\alpha}+1)(1+\hat{\beta})}{\Gamma(\hat{\alpha})} = covg(2)/covg(1)$$

$$\hat{\beta} = \frac{covg(2)\Gamma(\hat{\alpha})}{covg(1)\Gamma(\hat{\alpha}+1)} - 1$$

Finally:

$$p(0|\hat{\alpha},\hat{\beta}).c_0 = covg(1)$$

$$c_0 = \frac{covg(1)}{(\hat{\beta}/(1+\hat{\beta}))^{\hat{\alpha}}}$$

$$= covg(1).(\hat{\beta}/(1+\hat{\beta}))^{-\hat{\alpha}}$$

Expected number of erroneous k-mers with coverage x is given by:

$$errcovg(x) = c_0 \times p(x-1|\hat{\alpha},\hat{\beta}) \tag{14}$$

2 Theoretical proofs

In this section we prove some properties of linked de Bruijn graphs. We make the assumption that the graph is constructed from input sequences without reading errors or coverage gaps and that reads satisfy Ukkonen's condition wherein the read length L is at least one base longer than the longest interleaved or triple repeat (Ukkonen *et al.*, 1992, Bresler *et al.*, 2013).

2.1 Notation

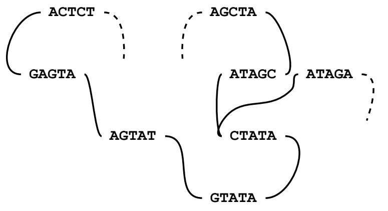

Figure 1: A portion of a de Bruijn graph with $k = 5$.

The Linked de Bruijn graph is defined as $LG(k) = (V, E, L)$ where k is the de Bruijn graph parameter, and V and E are defined as in a de Bruijn graph. Vertices V is the set of k-mer-keys. A k-mer is represented by a $\langle vertex, orientation\rangle$-tuple called an *oriented-vertex*. Oriented-vertex v' is vertex $v \in V$ with an orientation – either forwards $\overrightarrow{v}$ or backwards $\overleftarrow{v}$. $\widehat{v'}$ is the opposite orientation of v'. E is the set of directed edges between oriented-vertices. $L(v')$ is a set of valid paths through the graph (*links*) that start at oriented vertex $v' \in V'$.

Traversal means moving through oriented vertices and along edges between vertices, using link information. $v'_a \rightsquigarrow v'_b \rightsquigarrow v'_c$ means we start walking at vertex v'_a reach vertex v'_v then continue traversal to reach vertex v'_c, each with an unambiguous route. In other words if we start at v'_a there is only one valid path to follow and it reaches v'_b and so on to v'_c. $v'_v \not\rightsquigarrow v'_y$ means we cannot traverse unambiguously to vertex v'_y if we start at v'_x. We can therefore make the following statements:

$$v'_x \rightsquigarrow v'_y \rightsquigarrow v'_z \implies (v'_x \rightsquigarrow v'_y) \land (v'_x \rightsquigarrow v'_z) \tag{15}$$

$$v'_x \rightsquigarrow v'_y \not\Longrightarrow v'_y \rightsquigarrow v'_x \tag{16}$$

In Supplementary Figure 1, we can see that it is possible to traverse unambiguously from k-mer $AGCTA$ to $GAGTA$ ($\overrightarrow{AGCTA} \rightsquigarrow \overleftarrow{GAGTA}$) but not in the reverse direction ($\overrightarrow{GAGTA} \not\rightsquigarrow \overleftarrow{AGCTA}$). This is because we hit a fork (bifurcation) in the graph that cannot be resolved.

An oriented vertex in the graph represents a k-mer which occurs in one or more locations in the input sequences. For a given position in the input sequence s_x, we make the following definitions:

1. $V(s_x)$ is the oriented vertex representing the k-mer starting at position s_x in the input sequence; this is a many-to-one mapping.
2. $\widehat{V(s_x)}$ is $V(s_x)$ in the opposite orientation.
3. $S(v'_x)$ is the set of sequence positions represented by oriented vertex v'_x.

2.2 Repeats

A repeated substring is a substring that occurs more than once in the input genome. Repeated substrings may be overlapping and may occur reverse-complemented. A maximal repeated substring is a substring such that adding a single character to the head or tail would decrease the number of occurrences of it. A dynamic programming solution can find the set of maximal repeated substrings (including reverse-complements) for any string in time $O(N^2)$ and memory $O(N)$. As an

example, for the string *abababaa* the set of maximal repeated substrings is $\{abababa, ababa, aba, a\}$ with occurrence counts $\{2, 3, 4, 5\}$ respectively.

We can paint repeats onto the input genome by exhaustively finding all maximal substrings that appear at one or more other positions in the input sequence. Repeats must be of length $\geq k$, where k is the parameter of the de Bruijn graph. Graph construction is then equivalent to gluing all repeats together (see Supplementary Figure 2).

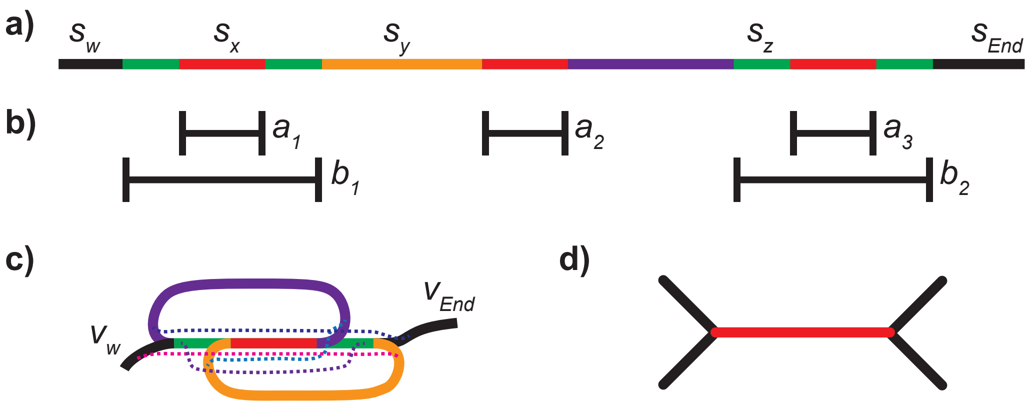

Figure 2: Repeats in an input sequence. a) input sequence, repeats highlighted, points s_w, s_x, s_y and s_z labelled b) repeats labelled c) graph structure with links added (dashed lines) d) general structure of all repeats in the graph

All repeats now take the general form shown in 2.(d) – at the start the graph collapses down from two or more vertices and at the end forks into two or more vertices. Between the start and end of the repeat the sequences that carry the repeat do not separate. We can now see that every fork in the graph is the ending of a repeat, and conversely that every repeat ends at a fork. If we are traversing a path through the graph and hit a fork, either we started in the repeat or we passed the start of the repeat that ends at this fork.

$$REP_S(s_w) = \varnothing \qquad REP_V(V(s_w)) = \varnothing$$
$$REP_S(s_x) = (a_1, b_1) \qquad REP_V(V(s_x)) = (a_1, a_2, a_3, b_1, b_2)$$
$$REP_S(s_y) = \varnothing \qquad REP_V(V(s_y)) = \varnothing$$
$$REP_S(s_z) = (b_2) \qquad REP_V(V(s_z)) = (b_1, b_2)$$

Figure 3: Repeat sets of points s_w, s_x, s_y and s_z in Supplementary Figure 2

We define:

1. $REP_S(s_x)$ as the set of repeats that sequence position s_x is contained in.
2. $REP_V(v'_x)$ as the union of $REP_S(X)$ for all positions X in the sequence where the k-mer associated with vertex v'_x appears:

$$REP_V(v'_x) = \bigcup_{s \in S(v'_x)} REP_S(s) \qquad (17)$$

An example is shown in Supplementary Figure 3. Note that vertex orientation has no effect on the set of repeats a vertex is in:

$$REP_V(v'_x) = REP_V(\widehat{v'_x}) \tag{18}$$

Entering repeats presents no issue in assembly. Leaving repeats requires some information about current location(s) in the underlying sequence. This information must be stored *before* you enter a repeat. Picture starting graph traversal from the middle of the red repeat in Supplementary Figure 2.(c): once you reach the end of the red repeat, you cannot make a decision about where to go. If you were to start at v'_w, you can see that just before you enter each repeat, you pick up an annotation which enables you to resolve it.

Starting traversal from within a repeat is equivalent to walking multiple places in the input sequence at once, and only tracing the consensus sequence of the repeats (stopping at the end of the repeat).

2.3 Sequence Traversal

Assuming a Linked de Bruijn Graph constructed with complete information (no sequencing error, coverage gaps and all repeats contain by at least one read), we can prove the following rules about traversal:

Proposition 2.1 (Sequence Traversal)**.** *Let s_x, s_y be points on the same input sequence, where s_y follows s_x. Traversal from the vertex representing s_x to the vertex representing s_y is possible $\iff$ the set of maximal substrings of s_x is a subset of s_y i.e.*

$$V(s_x) \rightsquigarrow V(s_y) \iff REP_S(s_x) \subseteq REP_S(s_y) \tag{19}$$

Proof. In order to traverse from one vertex to another we must not leave any of the repeats we were originally within, as doing so would mean we hit a fork we could not resolve, therefore:

$$V(s_x) \rightsquigarrow V(s_y) \implies REP_S(s_x) \subseteq REP_S(s_y) \tag{20}$$

If we enter repeats that start after s_x then we we pick up annotations just before they start which are used to resolve them. The only graph features that will stop traversal between two connected vertices are forks representing the end of repeats that we started within. These cannot be resolved unambiguously. Therefore if we do not leave repeats we started in, we can traverse connected vertices:

$$REP_S(s_x) \subseteq REP_S(s_y) \implies V(s_x) \rightsquigarrow V(s_y) \tag{21}$$

We can see that Proposition (2.1) follows from equations (20) and (21). □

Proposition 2.2 (Sequence Transitivity)**.** *Let s_x, s_y, s_z be points that appear in that order on the same input sequence. If we can start at $V(s_x)$ and reach $V(s_y)$ and start at $V(s_y)$ and reach $V(s_z)$, then if we start at $V(s_x)$ we can reach $V(s_z)$ i.e.*

$$(V(s_x) \rightsquigarrow V(s_y)) \land (V(s_y) \rightsquigarrow V(s_z)) \implies V(s_x) \rightsquigarrow V(s_z) \tag{22}$$

Proof.

$$\begin{aligned}
V(s_x) \rightsquigarrow V(s_y) &\implies REP_S(s_x) \subseteq REP_S(s_y) && \text{using (2.1)} && (23)\\
V(s_y) \rightsquigarrow V(s_z) &\implies REP_S(s_y) \subseteq REP_S(s_z) && \text{using (2.1)} && (24)\\
\text{Thus by (23) and (24)} &\implies REP_S(s_x) \subseteq REP_S(s_z) && &&\\
&\implies V(s_x) \rightsquigarrow V(s_z) && &&
\end{aligned}$$

□

2.4 Vertex Traversal

We define $REPEND_V(v'_x)$ to be the set of last oriented-vertices of repeats that vertex v'_x is in (see example in Supplementary Figure 4). The last vertex of a repeat is dependent on vertex orientation: $REPEND_V(v'_x)$ is not necessarily equal to $REPEND_V(\widehat{v'_x})$.

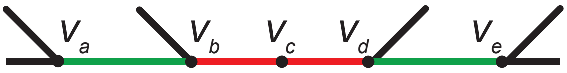

Figure 4: Illustration of $REPEND_V(\overrightarrow{v_x})$ function: $REPEND_V(\overrightarrow{v_c}) = (\overrightarrow{v_d}, \overrightarrow{v_e})$; $REPEND_V(\overleftarrow{v_c}) = (\overleftarrow{v_a}, \overleftarrow{v_b})$

$REPEND_V(v'_x)$ is a set of vertices that mark forks in the graph. The set of all fork vertices in the graph is:

$$\bigcup_{v \in \mathcal{V}} (REPEND_V(\overrightarrow{v}) \cup REPEND_V(\overleftarrow{v})) \tag{25}$$

where $\mathcal{V}$ is the collection of all vertices in the graph.

If you start traversal in a repeat it is not possible to leave it. That means if you start traversal at some vertex v'_x, you cannot traverse unambiguously past any vertex in $REPEND_V(v'_x)$.

Proposition 2.3 (Vertex Traversal)**.** *Let v'_1 and v'_m be vertices with orientations and $\{v'_1, \ldots, v'_n\}$ be a connected path through the graph. We can traverse starting at vertex v'_1 and reach vertex v'_n $\iff$ if none of the vertices $v'_1 ... v'_{n-1}$ are the last vertex of a member of $REP_V(v'_1)$ i.e.*

$$v'_1 \rightsquigarrow v'_n \iff v'_i \notin REPEND_V(v'_1) \quad \forall i \in \{1, 2, \ldots, n-1\} \tag{26}$$

Proof. By the same logic as Proposition (2.1). The only graph features that will stop traversal between two connected vertices are forks representing the end of repeats that we started within. If we can traverse from vertex v'_1 to vertex v'_n then repeats that started in v'_1 do not end before v'_n:

$$v'_1 \rightsquigarrow v'_n \implies v'_i \notin REPEND_V(v'_1) \quad \forall i \in \{1, 2, \ldots, n-1\} \tag{27}$$

If a repeat that we started in does not end before v'_n, then the only forks we encounter are from repeats that start and end between v'_1 and v'_n. For these repeats we have an opportunity to pick up annotations that will resolve them, therefore traversal will succeed:

$$v'_i \notin REPEND_V(v'_1) \quad \forall i \in \{1, 2, \ldots, n-1\} \implies v'_1 \rightsquigarrow v'_n \tag{28}$$

We cannot traverse past the last vertex of a repeat that we were already in when we started traversal of the graph. If we hit a vertex that is in $REPEND_V(v'_x)$, it marks a fork in the graph that we cannot resolve. We can see that Proposition (2.3) follows from equations (27) and (28). □

2.5 Lossless property

If we construct an annotated graph from a single sequence that starts and ends with a unique k-mer, following from Proposition (2.3), we are able to recover it in its entirety from the graph.

In addition to assuming error free coverage, we also assume that chromosomes start and end with unique k-mers. An undesired edge effect can appear if sequences end with k-mers that appear elsewhere in the graph, resulting in loops at the start or end of the graph representation of the sequences with in-degree greater than out-degree (see Supplementary Figure 5). You can force this to be true by explicitly adding a unique k-mer to the start/end of sequences. In the case of high coverage genome assembly this edge case is rare.

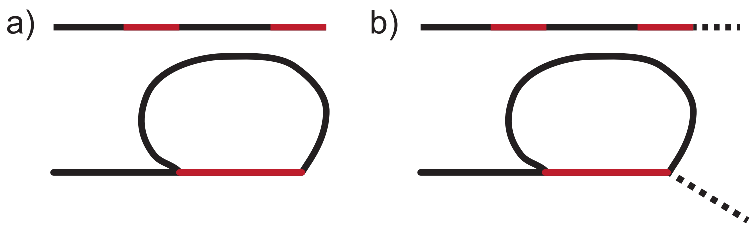

Figure 5: a) graph of sequence that ends with a repeat (in red) b) adding a unique sequence to the end of the input ensures infinite loops do not exist.

To extract exactly one contig from such a Linked de Bruijn graph we extract contigs and remove contained contigs (contigs that are substring, or reverse complemented substrings of other contigs).

3 Pipeline commands

Below, we provide the command listings used to produce assemblies on single-end data and paired-end data. Parameters and typical settings are indicated with a ${...[=X]} sigil (e.g. ${threads=8}) and remain fixed throughout the pipeline. Inputs/outputs are indicated with angle brackets with suggested file extensions (e.g. <build.ctx>).

3.1 McCortex pipeline

Assembly with McCortex consists of several steps encompassing initial construction of the de Bruijn graph (`build`), removal of sequencing errors (`clean`), addition of missed edges for $k-1$ overlaps (i.e. from reads that overlap by exactly $k-1$ bases) (`inferedges`), link construction from single- and paired-end reads (`thread`), link-informed contig emission (`contigs`), and contained contig removal (`rmsubstr`). The following pipeline listing demonstrates the use of these tools in sequence. Note that for read error correction and contig deduplication, we use existing tools `bfc` (Li, 2015) and `cd-hit-est` (Fu *et al.*, 2012).

```
# Error-correct reads
> bfc -s 3g -t16 <fastq_end_1.fq.gz> | gzip -1 > <corrected_1.fq.gz>
> bfc -s 3g -t16 <fastq_end_2.fq.gz> | gzip -1 > <corrected_2.fq.gz>

# Build a raw graph from fastq data with kmer size ${kmer_size},
# sample name ${sample_name}, and using maximum memory ${mem} (in gigabytes).
> mccortex63 build -m ${mem}G -k ${kmer_size} -s ${sample_name} \
                   -2 <corrected_1.fq.gz>:<corrected_2.fq.gz> <build.ctx>

# Remove sequencing errors using the Gamma-Poisson method.
> mccortex63 clean -m ${mem}G -o <clean.ctx> <build.ctx>

# Add edges between kmers that share k-1 bases.  These are edges that may
# have not been observed in the input data, but can be assumed to exist.
# This is important for ensuring proper graph connectivity for read threading.
> mccortex63 inferedges -m ${mem}G -o <infer.ctx> <clean.ctx>

# Pop bubbles in the graph
> mccortex63 popbubbles -f -m ${mem}G -o <popped.ctx> <infer.ctx>

# Thread single-ended reads through the graph to make links.
# If threading a different dataset (e.g. PacBio data) through this sample,
# this can be supplied in place of the original input fastq files.
> mccortex63 thread -m ${mem}G -t ${threads=8} \
                    -1 <corrected_1.fq.gz> -1 <corrected_2.fq.gz> \
                    -o <links_se.ctp.gz> <popped.ctx>

# Thread paired-end reads through the graph, with the help of connectivity
# information from the single-end links.
> mccortex63 thread -m ${mem}G -t ${threads=8} \
                    -2 <corrected_1.fq.gz>:<corrected_2.fq.gz> \
                    -p <links_se.ctp.gz> -o <links_pe.ctp.gz> <popped.ctx>

# Emit contigs using random seeds from around the graph.
> mccortex63 contigs -m ${mem}G -p <links_pe.ctp.gz> \
                     -o <contigs.fa> <popped.ctx>

# Remove redundant sequences from the contigs set with a
# sequence identity threshold of 95%
> cd-hit-est -M 4000 -c 0.95 -i <contigs.fa> -o <dedup.fa>
```

3.2 SGA pipeline

As we compare our workflow to SGA often in this manuscript, we have provided the program listing for our SGA-based pipelines. We used SGA version 0.10.15 for all analyses. Our pipeline is taken from the `sga-ecoli-miseq.sh` example script provided by the SGA software distribution, with the notable omission of the contig scaffolding step. For all analyses in this manuscript, only minor variations of this pipeline are required (in practice, we only modify the overlap value and the paired-end mode if we are working with single-end data).

```
# Preprocess data to remove ambiguous basecalls
> sga preprocess --pe-mode 1 -o <output.fq> \
                 <fastq_end_1.fq.gz> <fastq_end_2.fq.gz>

# Index reads
> sga index -a ropebwt -t ${threads=8} --no-reverse <output.fq>

# Perform error correction
> sga correct -k ${CK=41} --discard --learn -t ${threads} \
              -o <correct.fq> <output.fq>

# Index corrected reads
> sga index -a ropebwt -t ${threads=8} <correct.fq>

# Remove duplicates and reads with likely errors
> sga filter -x ${COV_FILTER=2} -t ${threads=8} --homopolymer-check \
             --low-complexity-check <correct.fq>

# Construct string graph
> sga overlap -m ${TAU_MIN=${OL}-5} -e ${EPSILON=0} ${threads=8} \
              <filter.fa>

# Assemble contigs
> sga assemble -m ${OL=47} -g ${MAX_GAP_DIFF=0} -r ${R=10} \
               -o ${assemble} <overlap>
```

3.3 SPAdes pipeline

Here we provide the command used for performing assemblies with SPAdes. In all analyses using SPAdes, we used version 3.11.1 of the software.

```
> spades.py -k ${kmer_size} --careful \
            -1 <fastq_end1.fq.gz> -2 <fastq_end2.fq.gz> \
            -o <output directory>
```

3.4 Velvet pipeline

For all assemblies using Velvet, we used the latest source code available from the Git repository (`https://github.com/dzerbino/velvet`, short commit hash: `9adf09f`). We ran Velvet in two modes: pure *de novo* assembly (i.e. using paired-end reads only), and reference-guided (using the Columbus module). In the latter case, our pipeline was:

```
> velveth <output directory> ${kmer_size} -shortPaired -fastq \
          -separate <fastq_end1.fq.gz> <fastq_end2.fq.gz>

> velvetg <output directory> -exp_cov 200 -ins_length_long 400 \
          -scaffolding no
```

For reference-guided assembly, we used the following pipeline:

```
> bwa mem <reference sequence> <fastq_end1.fq.gz> <fastq_end2.fq.gz> \
          > <output.sam>

> velveth <output directory> ${kmer_size} \
          -reference -fasta <reference sequence> \
          -shortPaired -sam <output.sam>

> velvetg <output directory> -exp_cov 200 -ins_length_long 400 \
          -scaffolding no
```

4 Number of links

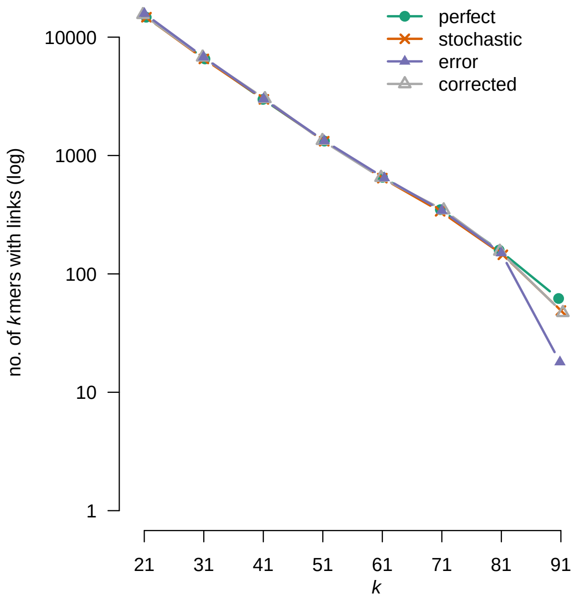

Figure 6: Number of k-mers with links as a function of k-mer size. Assembling 1 Mbp of sequence (human GRCh37 chr22:28,000,000-28,999,999) with three simulated 100X read data sets: (i) error free 100 bp reads, one read starting at every base (*"perfect"*); (ii) error free stochastic coverage, uniformly distributed read starts (*"stochastic"*); (iii) an error rate of 0.5% and stochastic uniformly distributed coverage (*"error"*); (iv) the *"error"* reads error-corrected with `bfc` (Li, 2015). Each graph has $\sim$ 1 million k-mers.

5 Variant calling in *K. pneumoniae*

We investigated the utility of links to call large variants (insertions or deletions greater than 100 bp in length). We obtained Illumina data (MiSeq 2×151 bp, $93X$ coverage; HiSeq 2×301 bp, $42X$) and PacBio RSII data (NCBI reference GCF_001870165.1_ASM187016v1) from a single haploid *K. pneumoniae* isolate, CAV1016. We constructed a dBG of the canonical reference sequence (NCBI reference GCF_000016305.1_ASM1630v1, $unisim 5.3 Mbp$) and the Illumina data for the study isolate at $k = 31$, omitting the PacBio data from graph construction for later use as validation. We then constructed an LdBG by using only the single-end reads from the study isolate for link construction. We implemented a sub program to find bubbles (graph motifs where paths diverge from a k-mer and rejoin at a later k-mer) and applied it first to the dBG, then to the LdBG, allowing events up to 200 kb in length. We removed events less than or equal to 100 bp in length, as well as duplicate events (arising from navigating the graph in both the forward and reverse direction). The reference and alternate alleles were validated by aligning each to the canonical reference and CAV1016 PacBio draft reference sequence, respectively. All alleles matched their respective sequences with 100% identity (0 mismatches, 0 gaps). Results are shown in Supplementary Table 1.

The filtered dBG and LdBG call sets contained 55 and 59 variants respectively. The variants exclusive to the LdBG call set consist of four insertions of lengths 134, 246, $7,952$ and $11,946$ bp (the corresponding rows in Supplementary Table 1 are highlighted). The dBG call set contained no exclusive variants.

Table 1: Large variant calls in *K. pneumoniae*, without and with links

	contig	pos	*without link information* type	length (ref) (bp)	length (alt) (bp)	*with link information* type	length (ref) (bp)	length (alt) (bp)
1	NC_009648.1	149901	ins	607	718	ins	607	718
2	NC_009648.1	591423	ins	317	1026	ins	317	1026
3	NC_009648.1	633960	del	8229	978	del	8229	978
4	NC_009648.1	666931	del	11764	11542	del	11764	11542
5	NC_009648.1	1158143	del	442	183	del	442	183
6	NC_009648.1	1163700	ins	176	1120	ins	176	1120
7	NC_009648.1	1405671	ins	637	5583	ins	637	5583
8	NC_009648.1	1494996	del	3967	1583	del	3967	1583
9	NC_009648.1	1541795	del	59808	1085	del	59808	1085
10	NC_009648.1	1542062	del	59541	818	del	59541	818
11	NC_009648.1	1558825	ins	548	656	ins	548	656
12	NC_009648.1	1564450	del	25853	20416	del	25853	20416
13	NC_009648.1	1597175	del	3162	1853	del	3162	1853
14	NC_009648.1	1617354	del	10408	446	del	10408	446
15	NC_009648.1	1714605	del	179	64	del	179	64
16	NC_009648.1	1809300	ins	289	9000	ins	289	9000
17	NC_009648.1	1894900	-	-	-	ins	1312	9264
18	NC_009648.1	1955922	del	2663	294	del	2663	294
19	NC_009648.1	1964316	del	3149	1525	del	3149	1525
20	NC_009648.1	1974462	del	4990	1283	del	4990	1283
21	NC_009648.1	1974510	del	5012	1305	del	5012	1305
22	NC_009648.1	2015329	del	782	453	del	782	453
23	NC_009648.1	2037377	del	1345	709	del	1345	709
24	NC_009648.1	2237525	del	1857	198	del	1857	198
25	NC_009648.1	2308668	-	-	-	ins	354	488
26	NC_009648.1	2522417	ins	1165	4427	ins	1165	4427
27	NC_009648.1	2656657	ins	199	556	ins	199	556
28	NC_009648.1	2691234	ins	491	4070	ins	491	4070
29	NC_009648.1	2728309	del	15624	11536	del	15624	11536
30	NC_009648.1	3135768	del	1215	524	del	1215	524
31	NC_009648.1	3628050	ins	1223	1643	ins	1223	1643
32	NC_009648.1	3803490	ins	462	930	ins	462	930
33	NC_009648.1	3816264	del	1985	1113	del	1985	1113
34	NC_009648.1	3826861	ins	2819	3608	ins	2819	3608
35	NC_009648.1	3831299	ins	620	2203	ins	620	2203
36	NC_009648.1	3863385	-	-	-	ins	478	12424
37	NC_009648.1	4037934	ins	449	630	ins	449	630
38	NC_009648.1	4143580	del	1355	559	del	1355	559
39	NC_009648.1	4169957	del	881	130	del	881	130
40	NC_009648.1	4323215	ins	442	1628	ins	442	1628
41	NC_009648.1	4361406	del	2963	1783	del	2963	1783
42	NC_009648.1	4362555	del	1814	634	del	1814	634
43	NC_009648.1	4501128	del	11949	397	del	11949	397
44	NC_009648.1	5020914	ins	3324	4623	ins	3324	4623
45	NC_009648.1	5035601	del	5026	864	del	5026	864
46	NC_009648.1	5035721	del	4906	744	del	4906	744
47	NC_009648.1	5061875	del	32222	118	del	32222	118
48	NC_009648.1	5091428	ins	454	6399	ins	454	6399
49	NC_009649.1	119259	ins	2596	7086	ins	2596	7086
50	NC_009649.1	132517	del	1510	405	del	1510	405
51	NC_009650.1	16509	ins	1122	11133	ins	1122	11133
52	NC_009650.1	19434	ins	1865	2418	ins	1865	2418
53	NC_009650.1	20210	del	1089	173	del	1089	173
54	NC_009650.1	23221	del	3232	1078	del	3232	1078
55	NC_009650.1	23297	del	1424	1071	del	1424	1071
56	NC_009651.1	35336	del	2322	67	del	2322	67
57	NC_009651.1	35337	-	-	-	ins	2987	3233
58	NC_009651.1	37623	ins	701	1543	ins	701	1543
59	NC_009651.1	77540	del	2054	560	del	2054	560

6 Links panel for *K. pneumoniae* isolate reconstruction

Table 2: Plasmids used for links panel in assembling 21 *K. pneumoniae* isolates with LdBG

ID	Plasmid	KPC allele	Length (bp)	Genbank accession	Ref
1	pKPC_UVA01	KPC-2	43,621	CP009465.1	Mathers *et al.* (2015)
2	pKPC_UVA02	KPC-2	113,105	CP009466.1	Mathers *et al.* (2015)
3	*E. coli* strain 233	KPC-3	10,192	JX500681.1	Roth *et al.* (2013)
4	pBK31567	KPC-5	47,387	JX193302.1	Chen *et al.* (2013)

In an effort to track plasmid transmission in a *K. pneumoniae* outbreak, Mathers *et al.* (2015) sequenced 37 isolates using the Illumina HiSeq 2000 platform, as well as generating draft reference genomes of two index case plasmid transformants with long reads from PacBio RSII instruments. Despite large homology between the two drafts (designated pKPC_UVA01 and pKPC_UVA02), mapping the Illumina reads to these sequences indicated that 21/37 isolates harbored KPC alleles on one of these two plasmid backgrounds. The authors were able to report a point mutation in isolate CAV1360's copy of KPC-2 (the altered allele being known as KPC-3). However, the alignments were insufficient to characterize a large alteration in CAV1077, nor could they detail other mutations upstream or downstream from the KPC gene.

We hypothesized that constructing a panel of links from plasmid sequences could enable reconstruction of the full plasmid sequences for the 21 isolates and permit us to describe the alterations more fully. The sequences included in the panel are listed in Supplementary Table 2.

In addition to the two Mathers *et al.* sequences, we included two others: a plasmid sequence from *E. coli* harboring KPC-3, and a plasmid sequence from an unrelated *K. pneumoniae* isolate harboring KPC-5. The *E. coli* sequence was chosen to be helpful for allele identification, but of limited utility for plasmid identification due to the divergent nature of its haplotypic sequence to the haplotypes present in the 21 *K. pneumoniae* isolates. The pBK31567 sequence was chosen as a negative control. As no isolates in our study carry the KPC-5 allele, this entry in the panel should go unutilized.

7 Performance metrics

To provide details on memory usage and performance for each step of a *de novo* assembly, and provide a baseline against which to evaluate these metrics, we computed runtime and memory usage of McCortex and SGA submodules on selected publicly available datasets. For our comparison, we chose *E. coli* (4.6 Mbp genome, $\sim 81x$ coverage), *P. falciparum* (23.3 Mbp, $\sim 63x$), and *C. elegans* (100.3 Mbp, $\sim 24x$). These results are summarized in Supplementary Table 3.

While both assemblers are designed to make fuller use of the connectivity information within reads, McCortex and SGA have vastly different design philosophies. SGA commands are designed to use very little memory, and across datasets, it is apparent that SGA uses a small fraction of the memory required by McCortex on the same dataset. McCortex attempts to balance memory usage with speed. All graph vertices and edges are loaded into memory upfront, while reads are processed in streaming (and parallelizable) fashion. Link construction also requires the storage of all putative links in memory until the full read dataset has been processed to ensure that support for each junction is correctly calculated. Thus, McCortex commands that store information based on reads supplied as input (`build` and `thread`) consistently have the highest memory use across the toolchain. The `clean` step requires as much memory as the `build` step in order to store the raw graph (containing genomic data and sequencing errors), but once most errors have been removed, memory usage by subsequent tools is reduced.

McCortex supports the use of multiple threads for processing data, greatly speeding up runtime. By default, the number of threads is 2 (one to read data from disk and one to perform processing steps). Link construction benefits from the use of many more threads, as the alignment of reads to the graph is an embarrassingly parallel process). As the major computational cost is in this `thread` step, our pipelines typically apply this step with several threads.

Table 3: Memory and runtime performance of McCortex and SGA across four datasets of varying size

			***E. coli* (4.6 Mbp)**		***P. falciparum* (23.3 Mbp)**		***C. elegans* (100.3 Mbp)**		***H. sapiens* (3,200 Mbp)**	
		Accession	ERR049156		ERR019061		ERR089806		ERR194147	
		Read length (bp)	75		76		100		101	
		Reads (×1e6)	5.0		19.4		24.5		787.3	
		Coverage	82x		64x		25x		25x	
**Command**	**Sub-command**	**Threads**	**Memory (GiB)**	**Time ([d:]hh:mm)**	**Memory (GiB)**	**Time ([d:]hh:mm)**	**Memory (GiB)**	**Time ([d:]hh:mm)**	**Memory (GiB)**	**Time ([d:]hh:mm)**
McCortex	*build*	2	2.1	0:02	3.1	0:12	7.2	0:20	220.4	12:23
	clean	2	1.6	0:01	2.9	0:02	7.0	0:07	212.2	3:45
	inferedges	2	0.1	0:01	0.4	0:01	2.1	0:03	59.4	1:55
	thread (single-end)	8 (32 for *H. sapiens*)	4.1	0:01	4.5	0:02	6.6	0:04	238.4	9:29
	thread (paired-end)	8 (32 for *H. sapiens*)	4.1	0:01	4.9	0:14	7.8	0:15	281.9	38:46
	contigs	2	0.2	0:01	0.7	0:01	3.5	0:03	127.8	3:16
	rmsubstr	2	5.4	0:01	1.4	0:01	4.8	0:03	270.5	30:11
	(max mem.; total time)		**4.1**	**0:08**	**4.9**	**0:33**	**9.3**	**0:55**	**281.9**	**4:03:45**
SGA	*preprocess*	1	< 0.1	0:01	< 0.1	0:03	< 0.1	0:04	-	-
	index	8	0.3	0:01	1.2	0:09	1.3	0:08	-	-
	correct	8	0.2	0:07	0.9	0:38	1.1	0:36	-	-
	index	8	0.3	0:04	1.1	0:17	1.2	0:16	-	-
	filter	8	0.3	0:03	1.5	0:22	1.2	0:17	-	-
	fm-merge	8	1.9	0:02	0.7	0:01	1.7	0:51	-	-
	index	8	< 0.1	0:01	0.7	0:01	1.9	0:07	-	-
	rmdup	8	< 0.1	0:01	< 0.1	0:01	0.2	0:02	-	-
	overlap	8	< 0.1	0:01	0.1	0:20	0.2	0:09	-	-
	assemble	1	< 0.1	0:01	0.3	0:03	0.7	0:11	-	-
	(max mem.; total time)		***3.0***	***0:22***	***1.5***	***1:55***	***1.9***	***2:41***	**-**	**-**

References

Bresler,G. *et al.*(2013). Optimal assembly for high throughput shotgun sequencing. *BMC Bioinformatics*, **14 Suppl 5**, S18

Chen,L. *et al.* (2013). Complete Nucleotide Sequences of blaKPC-4- and blaKPC-5-Harboring IncN and IncX Plasmids From Klebsiella Pneumoniae Strains Isolated in New Jersey. *Antimicrobial Agents and Chemotherapy*, **57**(1), 269–276.

Fu,L. *et al.* (2012). CD-HIT: Accelerated for Clustering the Next-Generation Sequencing Data. *Bioinformatics* **28**(23), 3150–3152.

Li,H. (2015). BFC: Correcting Illumina Sequencing Errors. *Bioinformatics* **31**(17), 2885–2887.

Mathers,A.J. *et al.* (2015). Klebsiella Pneumoniae Carbapenemase (KPC) Producing K. Pneumoniae at a Single Institution: Insights Into Endemicity From Whole Genome Sequencing. *Antimicrobial Agents and Chemotherapy* **59**(3). 1656–1663.

Roth,A.L. *et al.* (2013). Effect of Drug Treatment Options on the Mobility and Expression of blaKPC. *Journal of Antimicrobial Chemotherapy* **68**(12): 2779–2785.

Ukkonen,E. *et al.* (1992) Approximate String-Matching with Q-Grams and Maximal Matches. *Theoretical Computer Science* **92**(1): 191–211.
